# Differential antigenic imprinting effects between influenza H1N1 hemagglutinin and neuraminidase in a mouse model

**DOI:** 10.1128/jvi.01695-24

**Published:** 2024-12-05

**Authors:** Huibin Lv, Qi Wen Teo, Chang-Chun D. Lee, Weiwen Liang, Danbi Choi, Kevin J. Mao, Madison R. Ardagh, Akshita B. Gopal, Arjun Mehta, Matt Szlembarski, Roberto Bruzzone, Ian A. Wilson, Nicholas C. Wu, Chris K. P. Mok

**Affiliations:** 1 Carl R. Woese Institute for Genomic Biology, University of Illinois at Urbana-Champaign 14589 , Urbana, Illinois, USA; 2 Department of Biochemistry, University of Illinois at Urbana-Champaign 14589 , Urbana, Illinois, USA; 3 HKU-Pasteur Research Pole, School of Public Health, Li Ka Shing Faculty of Medicine, The University of Hong Kong 25809 , Hong Kong, China; 4 Department of Integrative Structural and Computational Biology, Scripps Research Institute 332494 , La Jolla, California, USA; 5 Centre for Immunology & Infection, Hong Kong Science Park 611449 , Hong Kong, China; 6 Istituto Pasteur Italia 204496 , Rome, Italy; 7 Skaggs Institute for Chemical Biology, The Scripps Research Institute 4356 , La Jolla, California, USA; 8 Center for Biophysics and Quantitative Biology, University of Illinois at Urbana-Champaign 14589 , Urbana, Illinois, USA; 9 Carle Illinois College of Medicine, University of Illinois Urbana-Champaign 14589 , Urbana, Illinois, USA; 10 The Jockey Club School of Public Health and Primary Care, The Chinese University of Hong Kong 569807 , Hong Kong, China; 11 Li Ka Shing Institute of Health Sciences, Faculty of Medicine, The Chinese University of Hong Kong 597321 , Hong Kong, China; 12 S.H. Ho Research Centre for Infectious Diseases, The Chinese University of Hong Kong 26451 , Hong Kong, China; 13 School of Biomedical Sciences, The Chinese University of Hong Kong 130379 , Hong Kong, China; St. Jude Children's Research Hospital, Memphis, Tennessee, USA

**Keywords:** immune history, hemagglutinin (HA), neuraminidase (NA), antigenic imprinting

## Abstract

Understanding how immune history influences influenza immunity is essential for developing effective vaccines and therapeutic strategies. This study examines the antigenic imprinting of influenza hemagglutinin (HA) and neuraminidase (NA) using a mouse model with sequential infections by H1N1 virus strains exhibiting substantial antigenic differences in HA. In our pre-2009 influenza infection model, we observed that mice with more extensive infection histories produced higher levels of functional NA-inhibiting antibodies (NAI). However, following further infection with the 2009 pandemic H1N1 strain, these mice demonstrated a reduced NAI to the challenged virus. Interestingly, prior exposure to older strains resulted in a lower HA antibody response (neutralization and HAI) to the challenged virus in both pre- and post-2009 scenarios, potentially due to faster viral clearance facilitated by immune memory recall. Overall, our findings reveal distinct trajectories in HA and NA immune responses, suggesting that immune imprinting can differentially impact these proteins based on the extent of antigenic variation in influenza viruses.

**IMPORTANCE:**

Influenza viruses continue to pose a significant threat to human health, with vaccine effectiveness remaining a persistent challenge. Individual immune history is a crucial factor that can influence antibody responses to subsequent influenza exposures. While many studies have explored how pre-existing antibodies shape the induction of anti-HA antibodies following influenza virus infections or vaccinations, the impact on anti-NA antibodies has been less extensively studied. Using a mouse model, our study demonstrates that within pre-2009 H1N1 strains, an extensive immune history negatively impacted anti-HA antibody responses but enhanced anti-NA antibody responses. However, in response to the 2009 pandemic H1N1 strain, which experienced an antigenic shift, both anti-HA and anti-NA antibody responses were hindered by antibodies from prior pre-2009 H1N1 virus infections. These findings provide important insights into how antigenic imprinting affects both anti-HA and anti-NA antibody responses and underscore the need to consider immune history in developing more effective influenza vaccination strategies.

## INTRODUCTION

During each influenza virus infection, the human immune system mounts a polyclonal antibody response targeting the two main surface glycoproteins of influenza virus: hemagglutinin (HA) and neuraminidase (NA). HA, the predominant surface antigen, consists of a globular head domain, which contains the receptor-binding site, and a stem domain that facilitates viral entry by mediating the fusion of viral and host membranes (1). In contrast, the NA protein aids in viral release by cleaving terminal sialic acids, allowing nascent virus particles to detach from the host cell membrane (2). Traditionally, it was thought that an effective humoral immune response to influenza virus was primarily driven by antibodies against HA. Consequently, influenza research has largely centered on HA, focusing on monoclonal antibody screening, functional epitope identification, and structural analysis. However, recent studies have demonstrated that anti-NA antibodies can also play a substantial antiviral role, independent of the HA antibody response (3–5).

The concept of original antigenic sin, introduced by Thomas Francis Jr. in the late 1950s, has evolved into what is now referred to as antigenic imprinting or antigenic seniority (6, 7). This phenomenon has been observed not only in influenza virus but also in other viruses, such as Dengue virus and SARS-CoV-2 (8–13). Since most individuals experience influenza infection during childhood and are subsequently re-exposed to antigenically drifted strains over time (14), antigenic imprinting suggests that immune history can influence both the magnitude and quality of antibody responses to subsequent infections (15, 16). A notable instance of this was observed during the 2009 H1N1 “swine flu” pandemic, where older individuals exhibited relatively lower mortality rates compared to younger age groups, likely due to their childhood exposure to antigenically similar H1N1 strains from the 1918 “Spanish flu” pandemic (17, 18). Despite these observations, the precise impact of immune history on antibody responses to the 2009 H1N1 pandemic virus remains poorly understood, partly due to the complexity of human infection and vaccination histories.

Given the abundance of HA on the influenza virus surface, antigenic imprinting has typically been studied in the context of anti-HA antibody responses (19–23). For example, early childhood infections with H1N1 or H3N2 influenza viruses are known to confer some level of protection against avian influenza strains like H5N1 and H7N9 later in life, likely due to cross-reactive anti-HA antibodies targeting conserved epitopes (24). However, the impact of antigenic imprinting on NA remains less well characterized (25, 26), and the effects of cumulative immune history on both HA and NA responses are still unclear.

In this study, we aim to mimic human immune history in a mouse model by sequentially infecting mice with up to four antigenically distinct influenza viruses, followed by a challenge with the 2009 H1N1 pandemic virus. We demonstrate that the extent of immune history can significantly influence the induction of both anti-HA and anti-NA antibodies. Furthermore, our findings suggest that the imprinting effects on HA and NA antibody responses differ after viral infection. These results underscore the importance of considering immune history in influenza vaccine design.

## RESULTS

### Establishment of a mouse model for sequential infections with heterologous influenza viruses

To model human sequential infections, we selected four pre-2009 H1N1 influenza strains: A/USSR/90/1977 (USSR/77), A/Chile/1/1983 (Chile/83), A/Beijing/262/1995 (Beijing/95), and A/Brisbane/59/2007 (Bris/07). These strains were chosen due to their historical roles as vaccine seed strains and their antigenic distinctions from one another (27). To minimize interference from the genetic background, we incorporated the HA and NA genes of each strain into the A/Puerto Rico/8/1934 (H1N1) backbone using the “6 + 2” reverse genetic approach (28).

**Fig 1:**
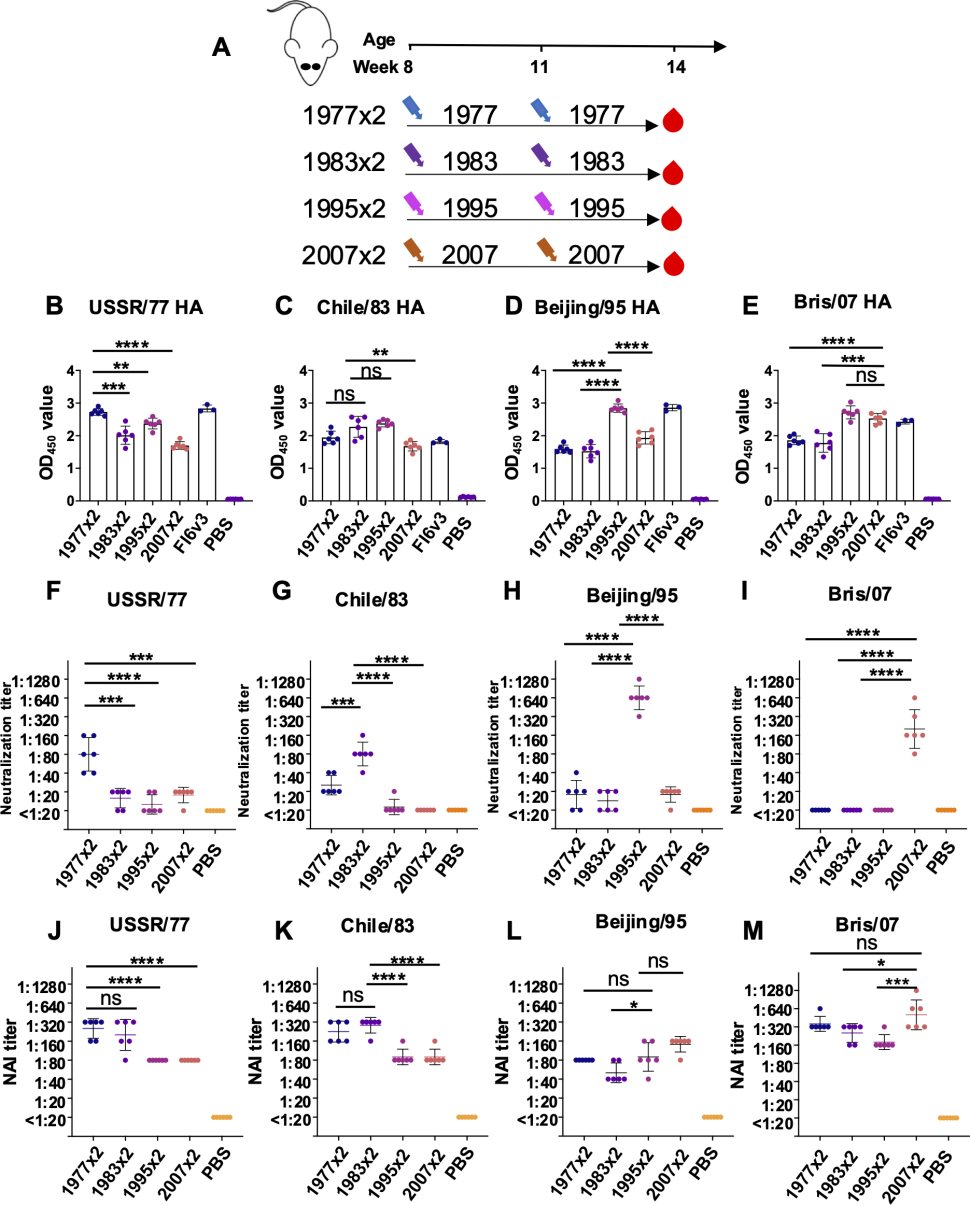
Binding, neutralizing, and NAI antibodies induced by sequential homologous viral infection. (**A**) Experimental design and sample collection. Six mice in each group were inoculated intranasally with a sequential homologous H1N1 virus infection strategy (1  ×  10^5^ PFU). (**B–E**) Binding antibodies against (**B**) USSR/77 HA, (**C**) Chile/83 HA, (**D**) Beijing/95 HA, and (**E**) Bris/07 HA were tested by ELISA. (**F–I**) Neutralizing antibodies against (**F**) USSR/77 virus, (**G**) Chile/83 virus, (**H**) Beijing/95 virus, and (**I**) Bris/07 virus were assessed by virus neutralization assay. (**J–M**) NAI antibody against (**J**) USSR/77 virus, (**K**) Chile/83 virus, (**L**) Beijing/95 virus, and (**M**) Bris/07 virus were measured by ELLA. Data are representative of two independent experiments performed in technical duplicate. FI6v3 is an influenza hemagglutinin stem-specific antibody, and PBS was used as a negative control. Error bars represent standard deviation. *P* values were calculated using a two-tailed *t*-test (^*^P < 0.05, ^**^P < 0.01, ^***^P < 0.001, ^****^P < 0.0001; ns, not significant).

Before conducting the sequential infection experiments, we assessed the cross-reactive antigenicity of both HA and NA from each virus. Eight-week-old BALB/c mice were sequentially infected with pairs of homologous viruses, administered 21 days apart. Plasma samples were then collected 21 days after the second infection (Fig. 1A). To evaluate binding and neutralizing capacities, we performed ELISA and microneutralization assays on each sample against all four H1N1 strains. The binding assays revealed cross-reactive antibodies to HA in the mice (Fig. 1B through E); however, cross-neutralization was limited, showing minimal neutralization against the three heterologous strains compared to the homologous strain (Fig. 1F through I). Interestingly, we observed strong cross-reactive NA inhibition (NAI) through enzyme-linked lectin assay (ELLA) across all groups (Fig. 1J through M), suggesting that antigenic drift in HA and NA may not occur synchronously (29, 30). This supports the hypothesis that immune responses to NA can remain cross-reactive even when HA antigenicity has drifted.

**Fig 2:**
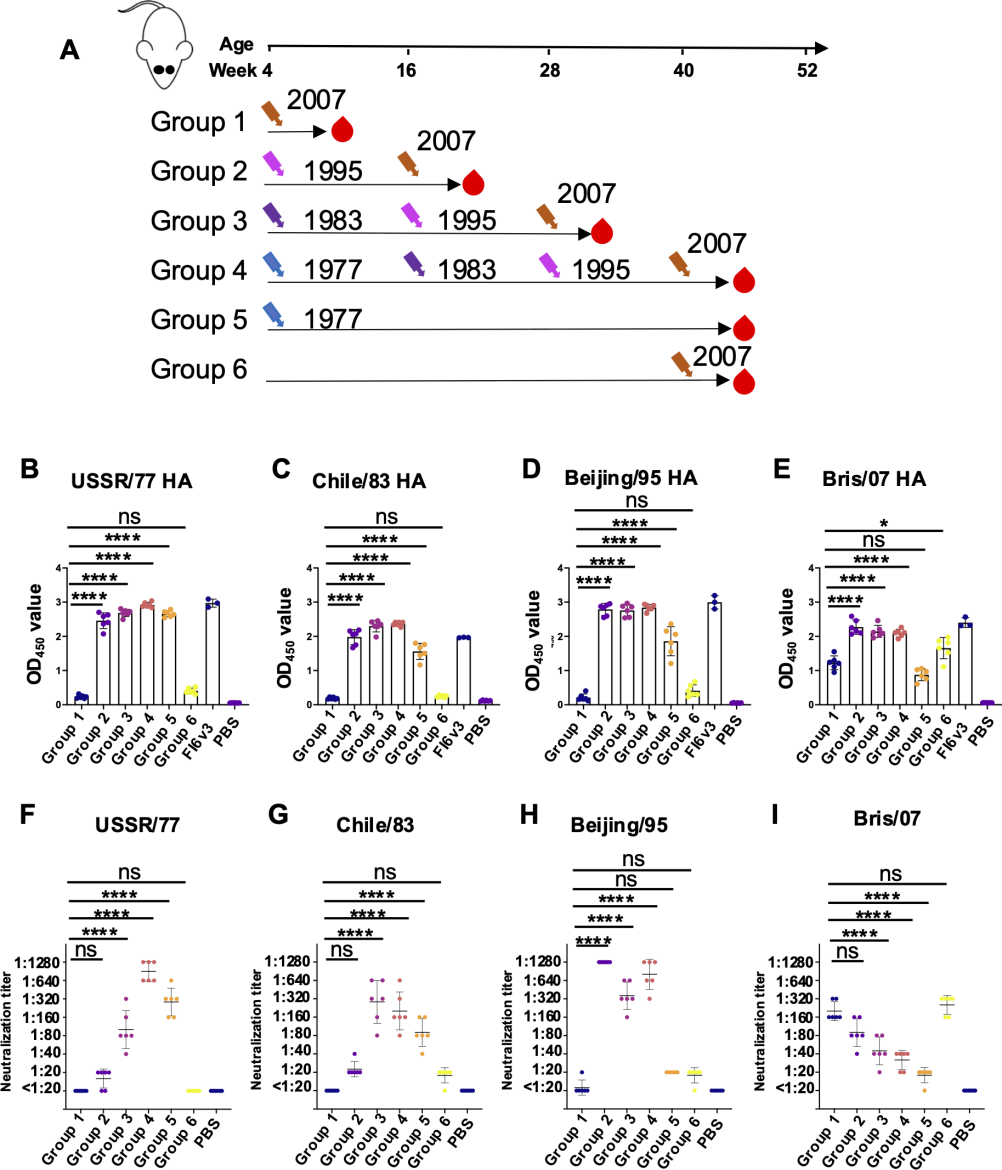
Binding and neutralizing antibodies after sequential viral infection. (**A**) Experimental design and sample collection. Six mice in each group were inoculated intranasally with a sequential H1N1 virus infection strategy (1  ×  10^5^ PFU). (**B–E**) Binding antibodies against (**B**) USSR/77 HA, (**C**) Chile/83 HA, (**D**) Beijing/95 HA, and (**E**) Bris/07 HA were tested by ELISA. (**F–I**) Neutralizing antibodies against (**F**) USSR/77 virus, (**G**) Chile/83 virus, (**H**) Beijing/95 virus, and (**I**) Bris/07 virus were assessed by virus neutralization assay. Data are representative of two independent experiments performed in technical duplicate. FI6v3 is an influenza hemagglutinin stem-specific antibody, and PBS was used as a negative control. Error bars represent standard deviation. *P* values were calculated using a two-tailed *t*-test (^*^P < 0.05, ^**^P < 0.01, ^***^P < 0.001, ^****^P < 0.0001; ns, not significant).

These findings set the stage for interpreting results from a more comprehensive experimental design involving sequential infection with different heterologous strains. Four-week-old BALB/c mice were divided into four groups: Group 1 was infected once with Bris/07; Group 2 underwent sequential infection with Beijing/95 followed by Bris/07, 12 weeks apart; Group 3 was sequentially infected with Chile/83, Beijing/95, and Bris/07, each 12 weeks apart; Group 4 experienced sequential infection with USSR/77, Chile/83, Beijing/95, and finally Bris/07, again 12 weeks apart (Fig. 2A). Two control groups were included: Group 5, infected once with USSR/77 and sampled after 39 weeks; and Group 6, comprising 40-week-old mice infected once with Bris/07. Plasma samples from all groups except Group 5 were collected 21 days post-last infection.

### Functional HA and NA antibodies show opposite trends following sequential infection with heterologous influenza viruses

To investigate antigenic imprinting, we tested the plasma samples for HA binding and neutralization against all four viruses (Fig. 2B through I). Sequential infection with heterologous H1N1 viruses generated cross-reactive binding antibodies to all four strains (*P* < 0.0001) (Fig. 2B through E). Notably, mice infected only with Bris/07 (Group 1) exhibited lower binding to their cognate HA protein compared to those that had prior exposure to heterologous viruses, while mice in Group 5, infected solely with USSR/77, developed cross-reactive binding antibodies to all four viruses (Fig. 2B through E), suggesting that exposure to earlier circulating strains can contribute to cross-reactivity with drifted viruses. This cross-reactivity, although slightly reduced compared to the parental virus, persisted for at least 43 weeks.

**Fig 3:**
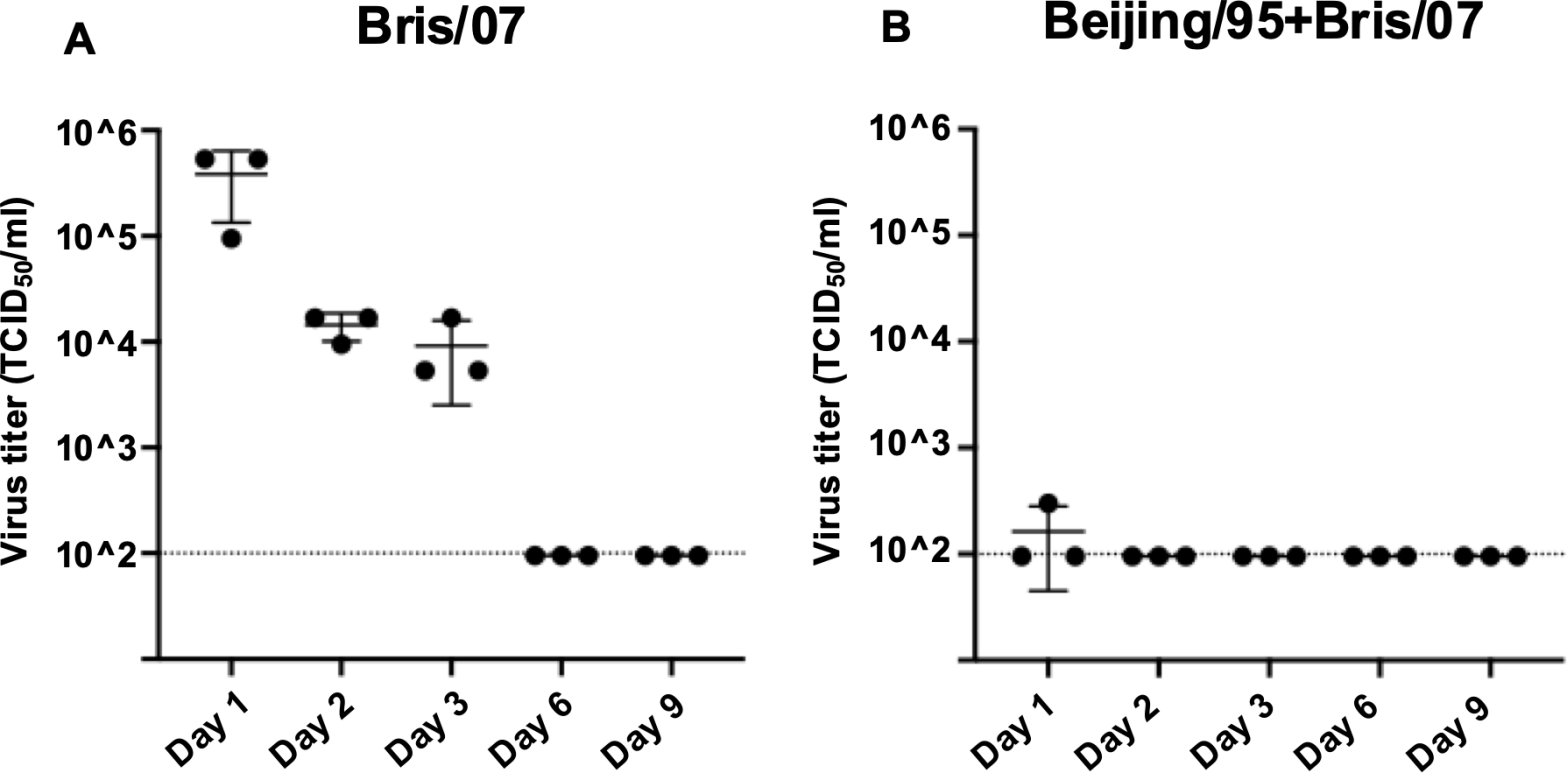
Lung viral titer after single Bris/07 infection and Beijing/95-Bris/07 sequential infection. Lung viral titers were measured on days 1, 2, 3, 6, and 9 after Bris/07 viral infection (**A**) and sequential Beijing/95-Bris/07 infection (**B**) (*n* = 3).

Conversely, the neutralizing activity against Bris/07 was highest in mice that had only been infected with this virus, without prior exposure to other strains (Groups 1 and 6). Neutralizing titers to Bris/07 decreased with an increasing number of sequential infections with other H1N1 strains (Fig. 2I). This trend suggests a potential relationship between immune priming and viral neutralization, where a more extensive history of prior infections may limit the production of neutralizing antibodies. To assess whether this association is due to limited viral replication resulting from recall memory, we compared lung viral titers between mice infected only with Bris/07 and those sequentially infected with Beijing/95 and then Bris/07. Lung samples were collected on days 1, 2, 3, 6, and 9 post-infection (Fig. 3). In group with Bris/07 only, lung viral titers progressively declined and were undetectable by day 6 (Fig. 3A). In contrast, viral levels in group with Beijing/95 and Bris/07 were nearly undetectable at all time points, indicating that prior immunity facilitated rapid viral clearance (Fig. 3B). These results suggest that the lower neutralizing antibody levels observed in previously infected mice could be due to the reduced antigen load in the lungs. Although Group 5 mice demonstrated relatively strong cross-reactive binding to Beijing/95 and Bris/07 (Fig. 2D and E), low neutralization was observed in the microneutralization assay (Fig. 2H and I), indicating that antibodies induced by USSR/77 infection may target non-neutralizing epitopes or possess relatively low affinity. Finally, a comparison of neutralizing antibody responses to Bris/07 between Groups 1 and 6 revealed similar immune responses in both young and elderly mice (Fig. 2I).

**Fig 4:**
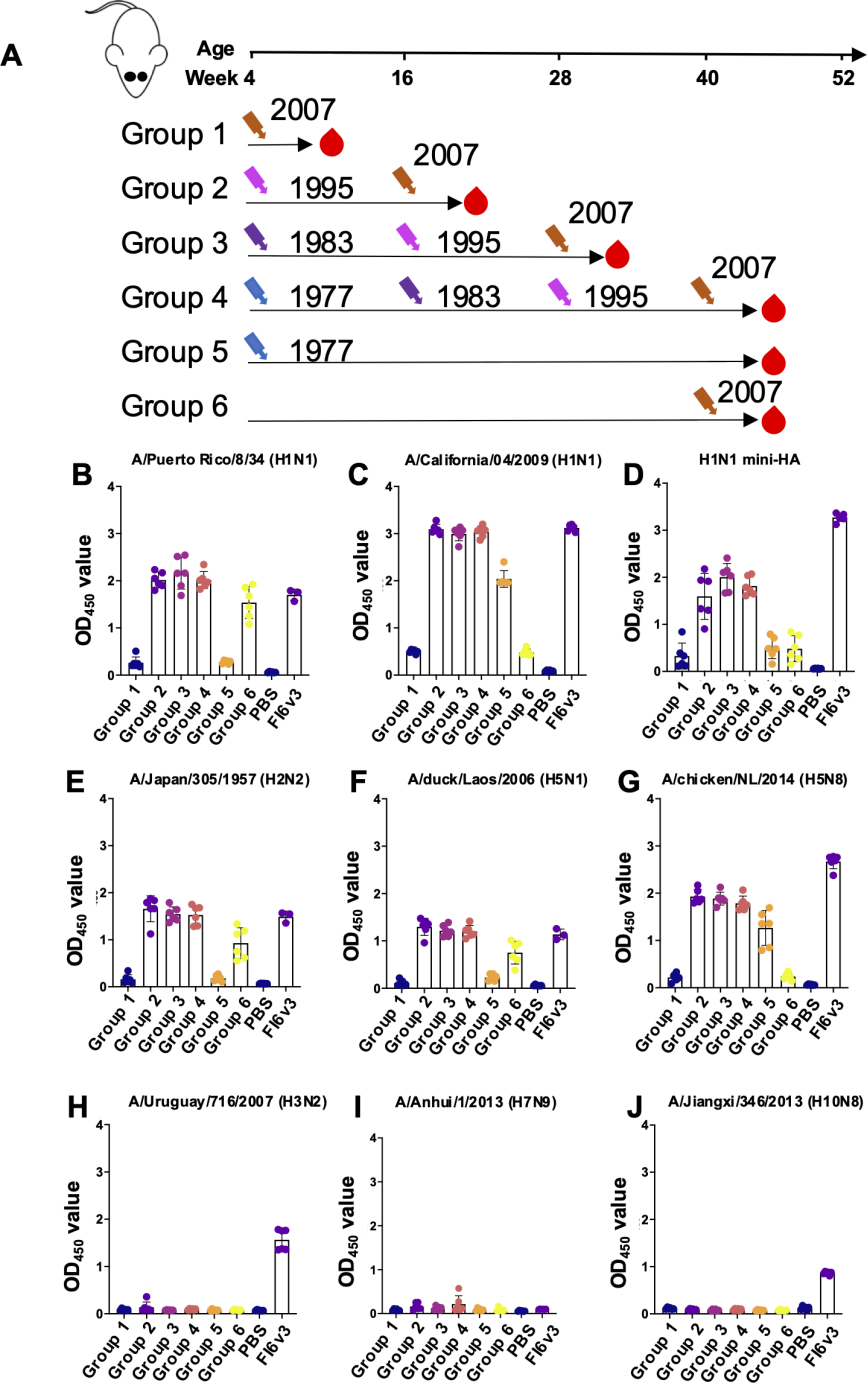
Cross-binding antibodies after sequential viral infection. (**A**) Experimental design and sample collection. Six mice in each group were inoculated intranasally with a sequential H1N1 virus infection strategy (1  ×  10^5^ PFU). (**B–J**) Binding antibodies against (**B**) A/Puerto Rico/8/34 (H1N1) HA, (**C**) A/California/04/2009 (H1N1) HA, (**D**) H1N1 mini-HA, (**E**) A/Japan/305/1957 (H2N2) HA, (**F**) A/duck/Laos/2006 (H5N1) HA, (**G**) A/chicken/NL/2014 (H5N8) HA, (**H**) A/Uruguay/716/2007 (H3N2) HA, (**I**) A/Anhui/1/2013 (H7N9) HA, and (**J**) A/Jiangxi/346/2013 (H10N8) HA were tested by ELISA. Data are representative of two independent experiments performed in technical duplicate. FI6v3 is an influenza hemagglutinin stem-specific antibody, and PBS was used as a negative control. Error bars represent standard deviation. *P* values were calculated using a two-tailed *t*-test (^*^P < 0.05, ^**^P < 0.01, ^***^P < 0.001, ^****^P < 0.0001; ns, not significant).

Influenza A viruses can be classified into two groups based on genomic differences in the HA protein. To further investigate cross-reactivity, we tested our samples for binding to a range of human and avian influenza viruses. Consistent with our findings in Fig. 2, we observed cross-reactivity against A/Puerto Rico/8/1934 (H1N1), A/California/07/2009 (H1N1), A/Japan/305/1957 (H2N2), A/duck/Laos/2006 (H5N1), and A/chicken/Netherlands/2014 (H5N8) in mice that had been infected with more than one virus (Fig. 4A through G). Using a mini-HA protein derived from the stem domain of Bris/07 (31), we found that this cross-reactivity may be attributed to the induction of stem-binding antibodies resulting from sequential infections (Fig. 4D). No cross-binding antibody responses were detected against A/Uruguay/716/2007 (H3N2), A/Anhui/1/2013 (H7N9), or A/Jiangxi/346/2013 (H10N8) (Fig. 4H through J), highlighting the specificity of these interactions and the antigenic distinctions within and between the two HA groups. Notably, the broad HA-binding antibody FI6v3 (32) was used as a positive control in these binding experiments.

**Fig 5:**
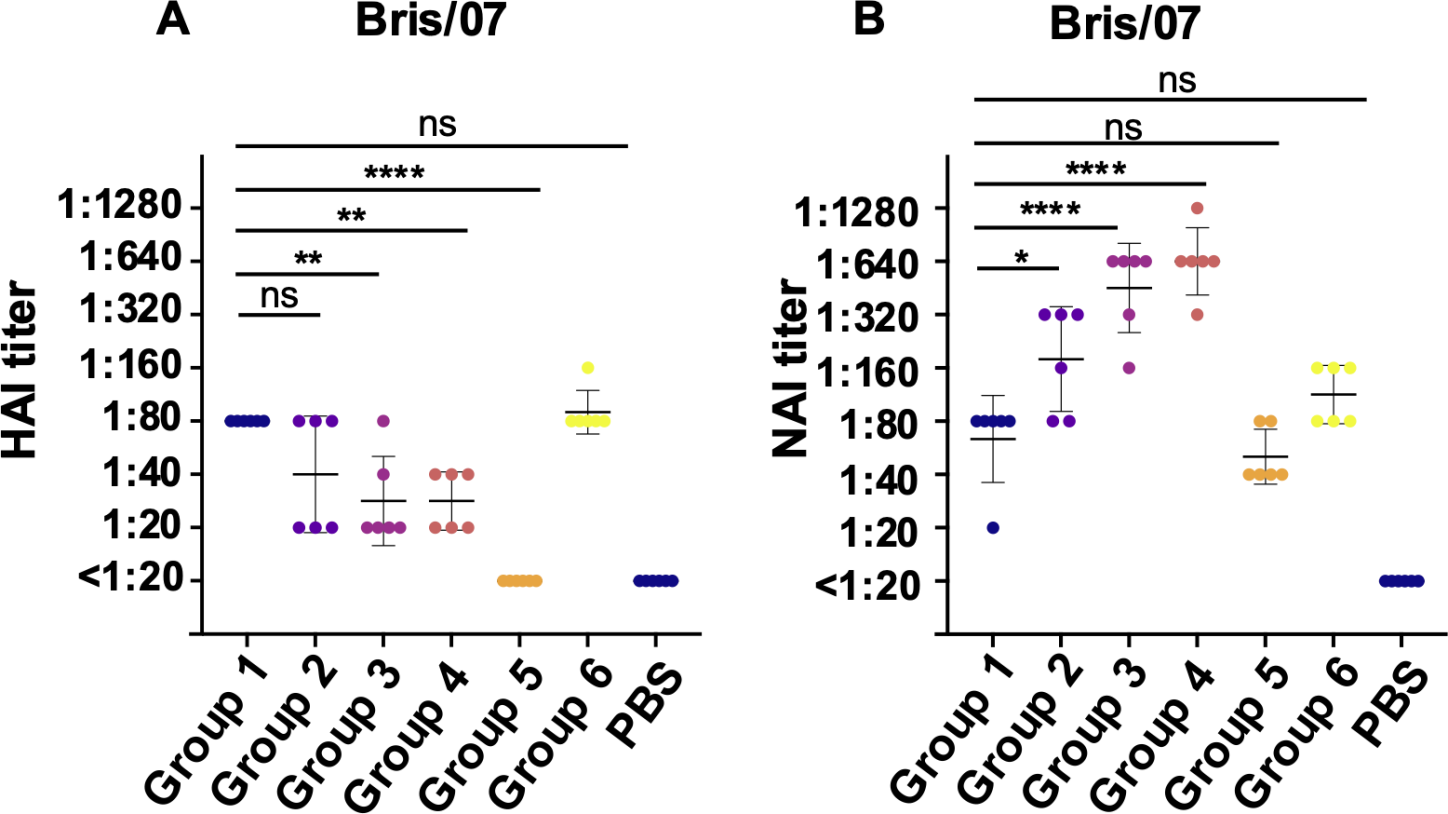
HAI and NAI antibodies after sequential viral infection. (**A**) Hemagglutination inhibiting antibody against Bris/07 H1N1 virus. (**B**) Neuraminidase inhibiting antibody against Bris/07 H1N1 virus. Data are representative of two independent experiments performed in technical duplicate. PBS was used as a negative control. Error bars represent standard deviation. *P* values were calculated using a two-tailed *t*-test (^*^P < 0.05, ^**^P < 0.01, ^***^P < 0.001, ^****^P < 0.0001; ns, not significant).

**Fig 6:**
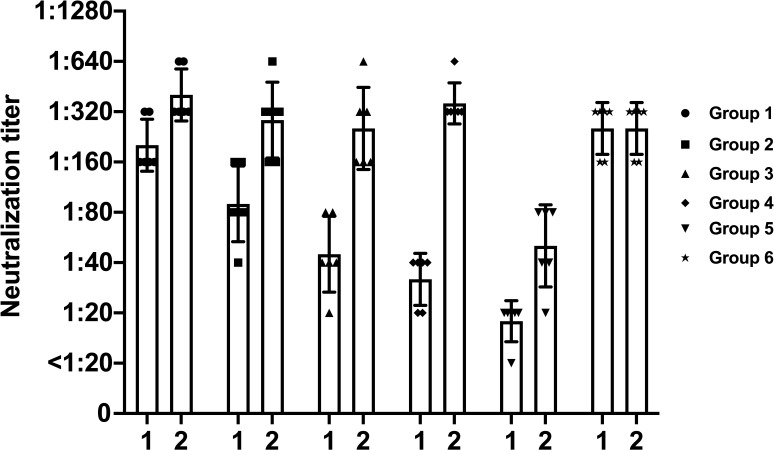
Anti-NA inhibiting antibodies after sequential viral infection against Bris/07 H1N1 virus. Anti-NA neutralizing antibodies against Bris/07 virus were assessed by virus inhibition assay. Data are representative of two independent experiments performed in technical duplicate. Error bars represent standard deviation. *P* values were calculated using a two-tailed *t*-test (^*^P < 0.05, ^****^P < 0.0001; ns, not significant).

While antigenic imprinting is typically associated with HA antibodies, the role of NA antibodies remains less understood. We examined both HA and NA responses using hemagglutination inhibition (HAI) and neuraminidase inhibition (NAI) assays. Mice previously infected with heterologous viruses showed lower HAI titers against Bris/07 compared to those infected only once with Bris/07 (Fig. 5A). In contrast, groups with a history of multiple infections exhibited higher functional NAI antibody titers (Fig. 5B). These data suggest that repeated exposures may boost antibody responses to conserved NA sites, reflecting an opposing pattern of immune response for HA and NA against the same virus. To further evaluate whether anti-NA antibodies contribute to viral replication inhibition, we replenished equal dilution of plasma instead of culture medium into corresponding wells after step of removing the “virus/plasma mixture” in a microneutralization assay (Fig. 6). Maintaining antibody concentrations in the culture media increased inhibitory activity against Bris/07 across Groups 1–4 (Fig. 6). These findings imply that anti-NA antibodies induced by infection may also play a significant role in reducing viral replication.

### Impact of antigenic shift on establishment of antigenic imprinting

**Fig 7:**
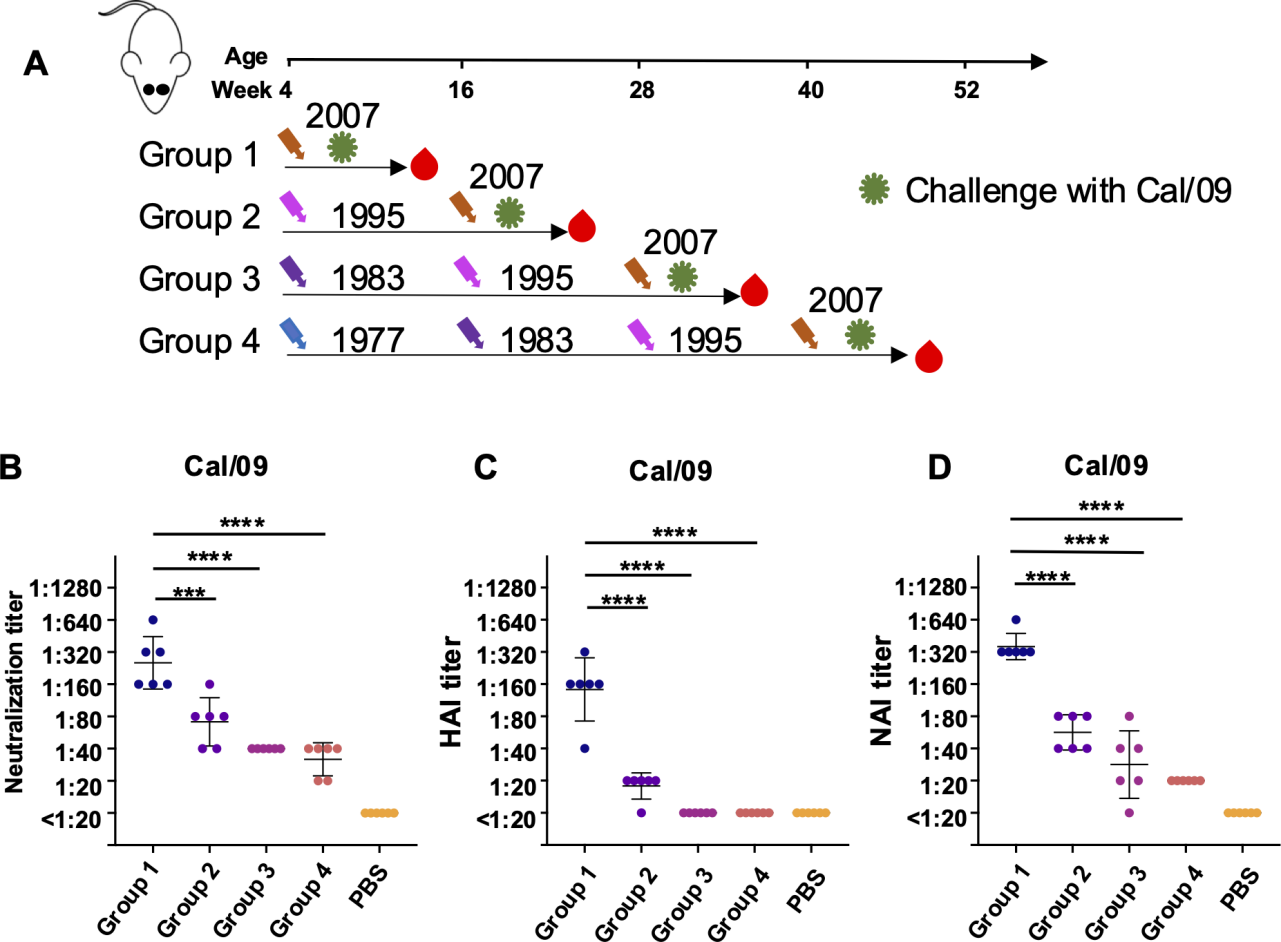
Neutralizing, HAI, and NAI antibodies with sequential infection history after Cal/09 H1N1 challenge. (**A**) Experimental design and sample collection. Six mice in each group were first inoculated intranasally with sequential H1N1 virus infection strategy (1  ×  10^5^ PFU) and were challenged with Cal/09 H1N1 virus (4  ×  10^5^ PFU). (**B**) Neutralizing antibodies against Cal/09 H1N1 virus were assessed by virus neutralization assay. (**C**) Hemagglutination inhibiting antibody against Cal/09 H1N1 virus. (**D**) Neuraminidase inhibiting antibody against Cal/09 H1N1 virus. Data are representative of two independent experiments performed in technical duplicate. PBS was used as a negative control. Error bars represent standard deviation. *P*-values were calculated using a two-tailed *t*-test (^*^P < 0.05, ^**^P < 0.01, ^***^P < 0.001, ^****^P < 0.0001; ns, not significant).

**Fig 8:**
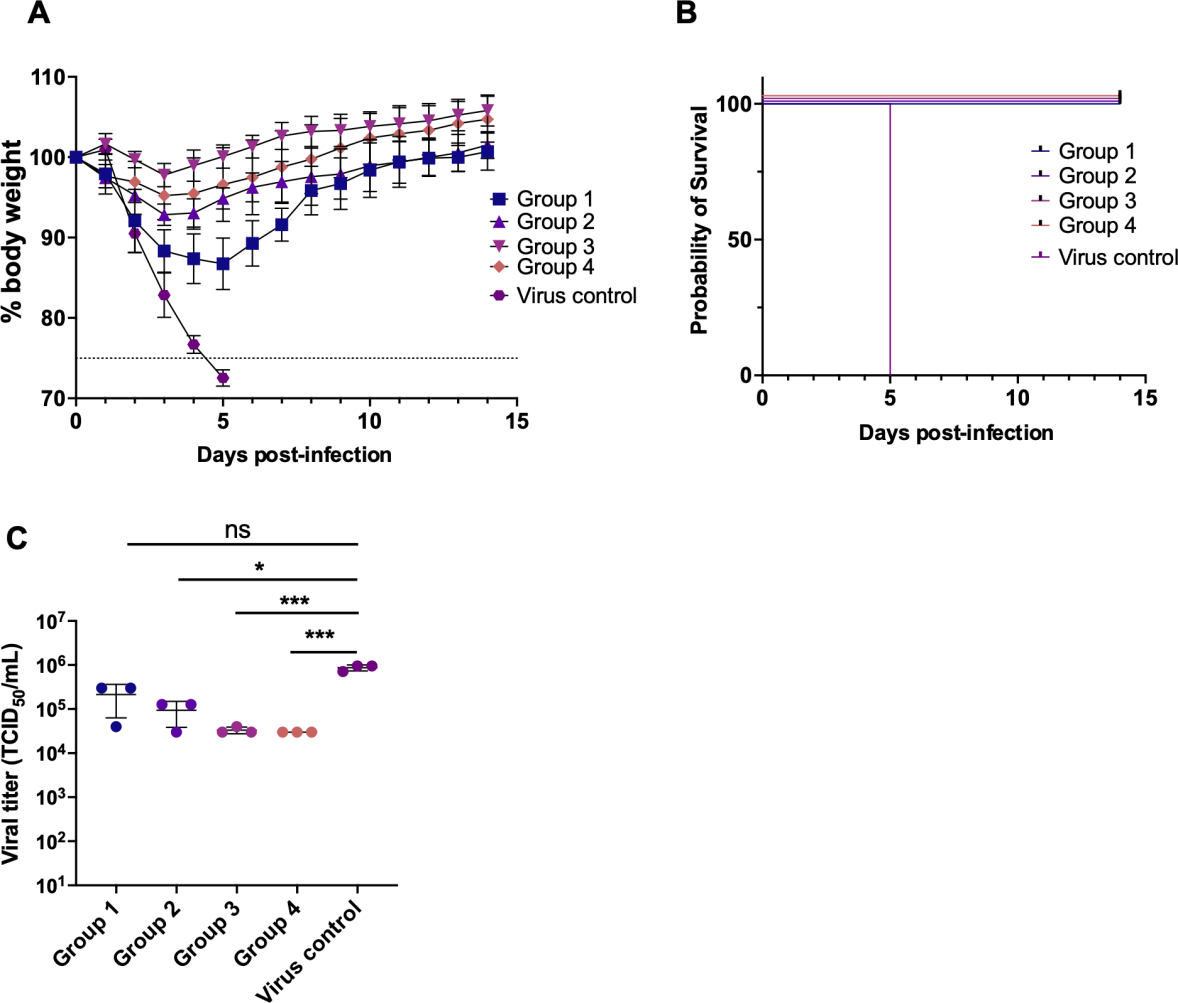
*In vivo* protection against Cal/09 H1N1 virus after sequential infection. (**A**) The mean percentage of body weight change post-infection is shown (*n* = 6). The humane endpoint, which was defined as a weight loss of 25% from initial weight on day 0, is shown as a dotted line. (**B**) Kaplan–Meier survival curves are shown (*n* = 6). (**C**) Lung viral titers on day 3 after infection are shown (*n* = 3). Solid black lines indicate means ± SD. *P* values were calculated using a two-tailed *t*-test (^*^P < 0.05, ^**^P < 0.01, ^***^P < 0.001, ^****^P < 0.0001; ns, not significant).

The swine-origin pandemic H1N1 virus, which underwent an antigenic shift from previous strains, has been the predominant strain since 2009. To explore the role of antigenic shift in the development of antigenic imprinting, we challenged Groups 1–4 mice with a lethal dose of Cal/09 (Fig. 7A). All previously infected mice demonstrated 100% protective efficacy, as evidenced by body weight recovery and survival (Fig. 8A and B). Notably, mice with more than two rounds of heterologous infection (Groups 2–4) showed a significant reduction in lung viral load compared to Group 1 (*P* < 0.05) (Fig. 8C). Interestingly, plasma collected 21 days after Cal/09 infection revealed not only reduced neutralization and HAI activity but also diminished NAI in mice with multiple heterologous infections, compared to those with only Bris/07 homologous infection (Fig. 7B through D). These results suggest that prior exposure to antigenically distinct strains can alter the immune response to a virus with a significant antigenic shift like Cal/09.

**Fig 9:**
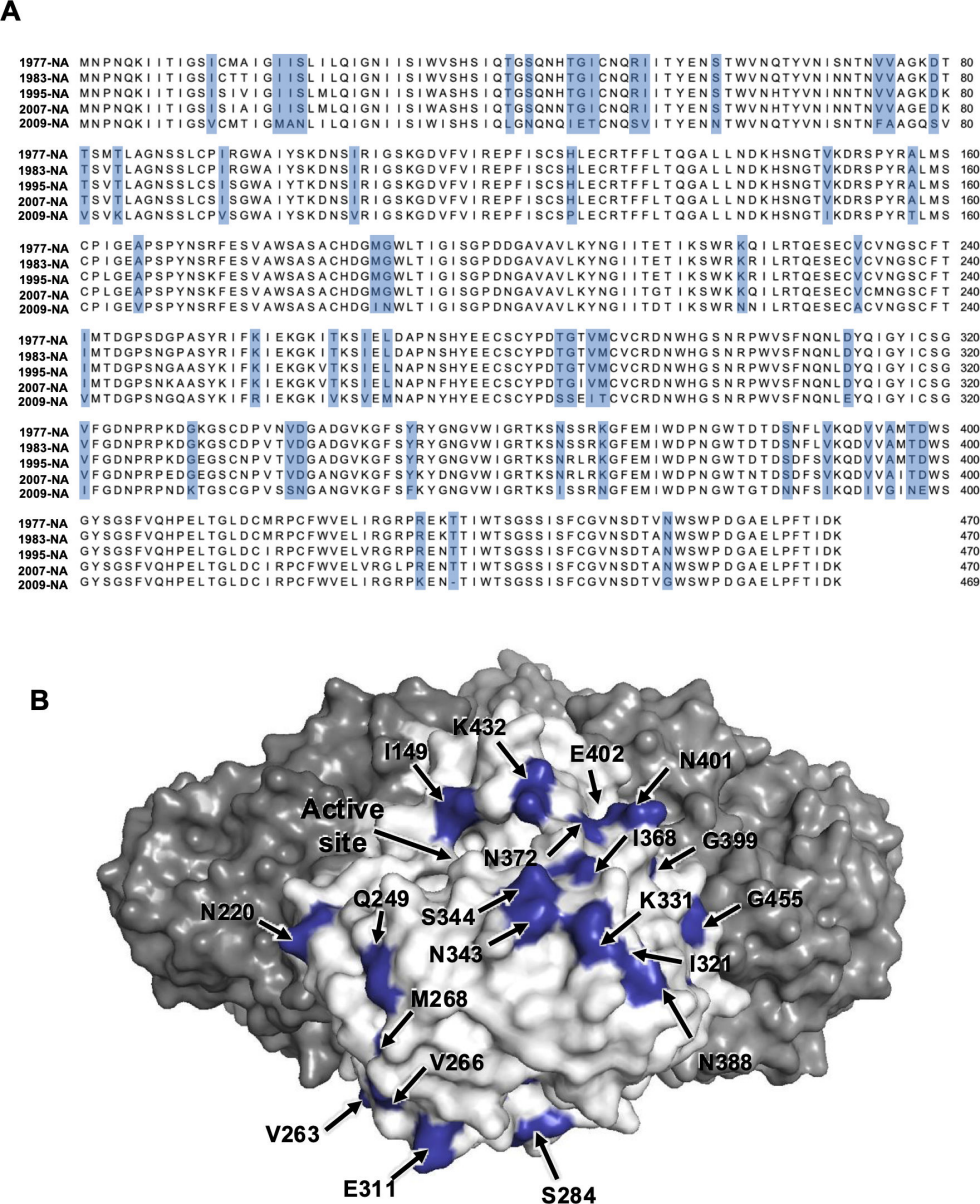
Surface residue difference among pre-2009 H1N1 NA and Cal/09 H1N1 NA. (**A**) Mutations are highlighted in blue on in a sequence alignment among four pre-2009 N1 protein and Cal/09 NA. (**B**) Surface residues on Cal/09 NA, which differs from four pre-2009 NAs, are highlighted on the Cal/09 NA protein.

To map the amino acid difference with Cal/09 NA, we compared amino acid residues in the NA of Cal/09 with those of the four pre-2009 H1N1 strains. We focused on amino acid residues that are completely conserved across the four pre-2009 NAs of interest but differed in Cal/09 NA (Fig. 9A). These residues are highlighted on the surface of Cal/09 NA structure (Fig. 9B). Many of these mutations surround the NA active site, such as I149, N220, Q249, K342, S343, N344, and N372. It is noted that most of these mutations are in the major antigenic sites for the NA protein (33). Moreover, several studies reported that some of the NA antibodies that bind outside the active site can inhibit NA activity by steric hindrance (3, 34). On the other hand, the glycosylation profiles have been also changed and may influence the antibody response in Cal/09 NA. For example, NWS at 455-457 in four pre-2009 N1 stains goes to GWS in Cal/09 N1 and 434 where it goes from KTT (1977 and 1983 N1) to NTT (glycan in 1995 and 2007 N1) to NTI (Cal/09). Taken together, NA antibodies induced by sequential infection of pre-2009 viruses in the mouse model may dominantly target the epitopes located in and around the active site that are conserved in pre-2009 strains but mutated in Cal/09. Therefore, these imprinted antibodies are escaped by Cal/09 virus. This observation further supports the notion that the antigenic disparity in the NA gene may contribute to antigenic imprinting following infection with the Cal/09 virus.

**Fig 10:**
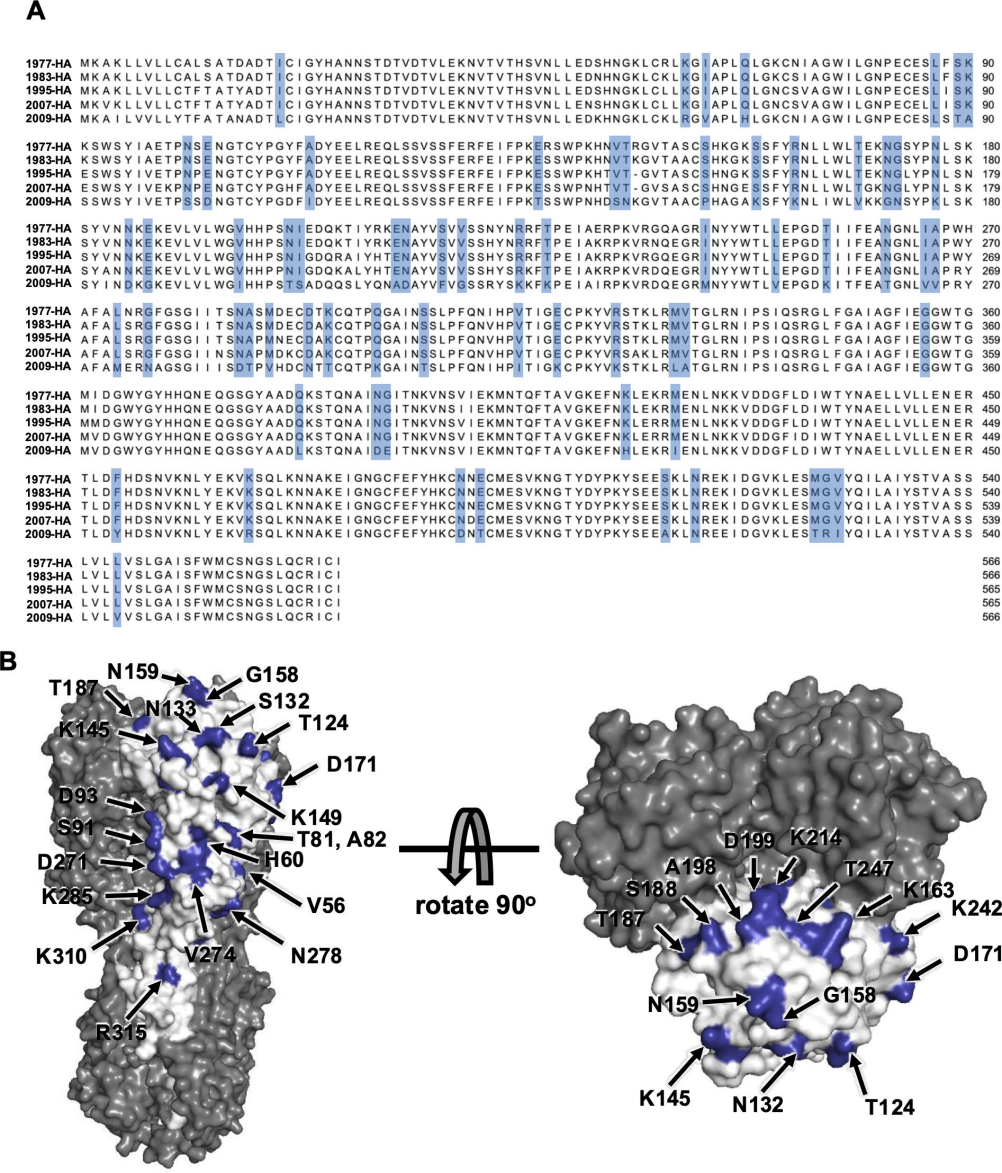
Surface residue difference among pre-2009 H1N1 HA and Cal/09 H1N1 HA. (**A**) Mutations are highlighted in blue in a sequence alignment among four pre-2009 HA protein and Cal/09 HA. (**B**) Surface residues on Cal/09 HA head domain, which differs from four pre-2009 HAs, are highlighted on the Cal/09 HA protein.

Similar analysis has been performed for pre-2009 and Cal/09 HA amino acid residues (Fig. 10A). Residues on the HA head domain are highlighted on the surface of Cal/09 HA structure (Fig. 10B). It is interesting that similar types of conserved residues are located close to the receptor binding site (K145, G158, N159, T187) among four pre-2009 stains, but we do not observe the same boosting effects in HA after sequential infection, as shown in the NA.

## DISCUSSION

In our study, we investigated the profile of immune response against influenza viruses by challenging mice with antigenic distinct strains sequentially. Our immunization strategy involved periodic, idealized exposure frequencies, while antibody titers and memory B cells were still close to peak levels (3 months from the previous exposures). This would be different from human exposures with likely much longer intervals between exposures. However, this model allowed us to capture immune dynamics under accelerated conditions and controllable settings, while the infection experience among human individuals is varied and difficult to identify. Nevertheless, the primary scope of our study is to address the questionwhether virus evolution may influence the establishment of immune imprinting in the host.

Interestingly, our data strongly support that antigenic imprinting on NA protein exists, but conclusion on HA is still uncertain. In our mouse model that mimics the pre-pandemic situation (before 2009) (Fig. 2), pre-exposure to antigenically distinct strains contributed to higher level of NAI to the Bris/07 upon infection. This can explain by the fact that there is a high structural similarity among different pre-pandemic H1N1 viruses that stimulate pre-existing B cell memory and enhance the production of functional NA antibodies against conserved epitopes at the enzymatic site of NA. Interestingly, when encountering the pandemic strain (Cal/09) in our model which the new virus has significant epitope changes, the pre-exposure was negatively impact the induction of NAI against Cal/09. In this situation, the pre-existing memory of those conserved but non-enzymatic sites in pre-exposure mice may become dominant. Our findings indicate that the influence of pre-existing memory on NA may differ in the context of subsequent viral infections.

The immune imprinting of HA in our model remains inconclusive. Despite a reduction of neutralizing antibody in the mice with more pre-exposure of other viruses upon the infection of Bris/07 or Cal/09 strain in our model, we found that the mice with previous infection showed faster virus clearance than those immune-naive mice (Fig. 3). The lower level and shorten duration of replication are possibly caused by the non-neutralizing antibodies or T cell response resulting to fewer HA antigens for triggering neutralizing antibodies. This protection mechanism that is caused by the recall memory may diminish the imprinting effect of HA.

Immune reprinting was also studied in other studies using ferret as a model. Previous research by O’Donnell et al. observed that ferrets with prior seasonal H1N1 infections did not show evidence of original antigenic sin when exposed to the 2009 pandemic H1N1 virus (35). Their study, focusing on antigenic imprinting on HA, employed a prime-boost strategy but did not further explore the influence of the extent of immune history on this phenomenon. Our study also found that a single prior exposure to a pre-2009 H1N1 strain does not show obvious imprinting response to the infection of Cal/09 strain, which is consistent with observations from the ferret study (35). The results from our model also provide additional information on how infection history affects antibody responses to NA.

Another notable aspect of our study is the implication of different immunodominant NA epitopes across various animal species. A study from Daulagala et al. showed lower cross-NAI activity in ferret sera after single H1N1 viral infection with virus strains between 1977 and 1991, while our mouse model displayed apparent cross-reactivity among NA strains from different years after repeated infection with the same virus (26). This discrepancy underscores the possibility of species-specific grouping of immunodominant NA epitopes, similar to a pattern also observable in HA. Liu et al. previously demonstrated that, in mice, the antigenic epitopes Sb and Cb2 are immunodominant, while ferret sera predominantly recognize antigenic epitope Sa (36). Validating the NA immunodominant epitopes and identifying the hierarchy of the NA immunodominant sites in humans could provide valuable information for the rational design of universal vaccines. Nevertheless, a prevalent subclade generally can circulate for a few years before it is replaced. Single or repeated infection to a particular strain may occur in humans.

In conclusion, our study offers substantial insights into the dynamics of the human immune response to influenza viruses, particularly to both HA and NA. It highlights how the extent of infection history influences antibody responses, a critical factor in the context of antigenic drift and shift. These findings have important implications for enhancing our understanding of influenza and for developing more effective vaccinations.

## MATERIALS AND METHODS

### Cells

HEK293T and MDCK cells were cultured in Dulbecco’s Modified Eagle’s Medium (high glucose; Gibco) supplemented with 10% heat-inactivated fetal bovine serum (Gibco), 1% penicillin-streptomycin (PS; Gibco), and 1% Gluta-Max (Gibco). Cells were passaged every 3–4 days using 0.05% Trypsin-EDTA solution (Gibco).

### Protein expression and purification

Mini-HA #4900 (31), A/Chile/1/1983 (H1N1) HA, A/Puerto Rico/8/1934 (H1N1) HA, and A/Japan/305/1957 (H2N2) HA proteins were fused with an N-terminal gp67 signal peptide and a C-terminal BirA biotinylation site, thrombin cleavage site, trimerization domain, and Hisx6 tag. These were then cloned into a customized baculovirus transfer vector. Recombinant bacmid DNA was generated using the Bac-to-Bac system (Thermo Fisher Scientific), following the manufacturer’s instructions. Baculovirus was produced by transfecting purified bacmid DNA into adherent Sf9 cells using Cellfectin reagent (Thermo Fisher Scientific), as per the manufacturer’s instructions. The baculovirus was amplified in adherent Sf9 cells at a multiplicity of infection (MOI) of 1. Recombinant proteins were expressed by infecting 1L of suspension Sf9 cells at an MOI of 1. After 3 days of post-infection, Sf9 cells were centrifuged at 4000 × *g* for 25 min, and soluble recombinant proteins were purified from the supernatant using Ni Sepharose excel resin (Cytiva), followed by size exclusion chromatography with a HiLoad 16/100 Superdex 200 prep grade column (Cytiva) in 20 mM Tris-HCl pH 8.0, 100 mM NaCl. Proteins were concentrated using an Amicon spin filter (Millipore Sigma) and filtered through 0.22-µm centrifuge Tube Filters (Costar). Protein concentration was determined by Nanodrop (Fisher Scientific), and proteins were aliquoted, flash-frozen in a dry-ice ethanol mixture, and stored at −80°C until use.

HA proteins A/Brisbane/59/2007 (H1N1) (NR-28607), A/California/04/2009 (H1N1) pdm09 (NR-15749), A/duck/Laos/3295/2006 (H5N1) (NR-13509), A/chicken/Netherlands/14015531/2014 (H5N8) (NR-50110), A/Uruguay/716/2007 (H3N2) (NR-15168), A/Anhui/1/2013 (H7N9) (NR-44081), and A/Jiangxi/346/2013 (H10N8) (NR-49440) were obtained from BEI Resources, NIAID, NIH (https://www.beiresources.org/).

### Recombinant virus construction and purification

H1N1 recombinant viruses A/USSR/90/1977 (HA, NA) × A/Puerto Rico/8/1934 (H1N1) (NR-3666), A/Chile/1/1983 (HA, NA) × A/Puerto Rico/8/1934 (H1N1) (NR-3585), A/Beijing/262/1995 (HA, NA) × A/Puerto Rico/8/1934 (H1N1) (NR-3571), and A/Brisbane/59/2007 (HA, NA) × A/Puerto Rico/8/1934 (H1N1) (NR-41797) were obtained from BEI Resources, NIAID, NIH. Recombinant viruses were constructed using a reverse genetics system, as previously described (28). Briefly, constructed HA and NA DNA plasmids were cloned and transfected into human embryonic kidney 293T cells (ATCC) and Madin-Darby canine kidney (MDCK) cells with a 6-segment plasmid encoding essential viral proteins and virus-like RNA of PR8. Supernatants were injected into 8–10-day-old embryonated chicken eggs for viral rescue at 37°C for 48 hours. Viruses were plaque-purified on MDCK cells grown in Dulbecco’s Modified Eagle’s Medium (Gibco) containing 10% fetal bovine serum (Gibco) and a penicillin-streptomycin mix (100 units/mL penicillin and 100 µg/mL streptomycin, Gibco). Individual plaques were picked and injected into embryonated eggs, and viral RNAs were extracted from allantoic fluids. HA and NA segments were confirmed by Sanger sequencing.

### Mouse infection and sample collection

BALB/c mice were anesthetized with ketamine and xylazine and intranasally infected with 10^5^ PFU of influenza virus, previously diluted in phosphate-buffered saline (PBS). Mouse plasma samples were collected in tubes containing heparin as an anticoagulant on day 21 post-infection. The experiments were conducted in the University of Hong Kong’s Biosafety Level 2 (BSL2) facility. The study protocol adhered strictly to the recommendations and was approved by the University of Hong Kong’s Committee on the Use of Live Animals in Teaching and Research (CULATR 5598-20).

### Enzyme-linked immunosorbent assay

Nunc MaxiSorp ELISA plates (Thermo Fisher Scientific) were coated overnight at 4°C with 100 µL of recombinant proteins at 1 µg/mL in 1 × PBS. On the next day, plates were washed three times with 1 × PBS containing 0.05% Tween 20 and blocked with 100 µL of ChonBlock blocking/sample dilution ELISA buffer (Chondrex Inc, Redmond, US) for 1 hour at room temperature. Plasma samples, diluted 1:100, were incubated for 2 hours at 37°C. Plates were then washed three times and incubated with horseradish peroxidase-conjugated goat anti-mouse IgG antibody (GE Healthcare) diluted 1:5,000 for 1 hour at 37°C. After five washes with PBS containing 0.05% Tween 20, 100 µL of 1-Step TMB ELISA Substrate Solution (Thermo Fisher Scientific) was added to each well. Following a 15-minute incubation, the reaction was stopped with 50 µL of 2 M H_2_SO_4_ solution, and absorbance was measured at 450 nm using a Sunrise (Tecan, Männedorf, Switzerland) absorbance microplate reader.

### Microneutralization assay

For the microneutralization (MN) assay, MDCK cells were prepared in each well of 96-well cell culture plates 1 day before the assay, ensuring a 100% confluent monolayer. Cells were washed once with phosphate-buffered saline (Gibco) and replaced with minimal essential media (MEM; Gibco) containing 25 mM HEPES (Gibco) and 100 U/mL penicillin-streptomycin (Gibco). All plasma samples for the MN assay were heat-inactivated at 56°C for 30 minutes. Twofold serial dilutions were performed on the heated plasma to create a dilution series ranging from 1:20 to 1:2560. These dilutions were mixed with 100 TCID_50_ of viruses in an equivalent volume and incubated at 37°C for 1 hour. The mixture was then inoculated into cells and incubated at 37°C for another hour. Cell supernatants were discarded and replaced with MEM containing 25 mM HEPES, 100 U/mL PS, and 1 µg/mL TPCK-trypsin (Sigma). Plates were incubated at 37°C for 72 hours, and virus presence was detected by a hemagglutination assay, with results recorded as the MN_50_ titer. To evaluate both anti-HA and anti-NA antibodies, we add the same dilution of the plasma samples back to well, instead of discarding the supernatants before incubation for 72 hours.

### Hemagglutination inhibition assays

Plasma samples were serially diluted twofold in a 96-well round-bottom plate in a total volume of 25 µL of phosphate-buffered saline. After dilution, 25 µL of virus (four hemagglutinating units) in PBS were added to each well and incubated for 30 minutes. Then, 50 µL of a 1.0% (vol/vol) solution of turkey erythrocytes was added, and the mixture was gently stirred. After 30 minutes at room temperature, the plates were read, and titers were determined as the lowest concentration of monoclonal antibody that fully inhibited agglutination. HAI assays were performed in duplicate.

### Enzyme-linked lectin assay

ELLA experiments were performed as described below. Briefly, each well of a 96-well microtiter plate (Thermo Fisher) was coated with 100 µL of fetuin (Sigma) at a concentration of 25 µg/mL in coating buffer (KPL coating solution; SeraCare) and incubated overnight at 4°C. On the following day, 50 µL of plasma samples at the indicated dilution in 2-(N-morpholino) ethanesulfonic acid (MES) buffer (pH 6.5), containing 20 mM CaCl2, 1% bovine serum albumin, and 0.5% Tween 20, were mixed with an equal volume of H1N1 virus. This mixture was added to the fetuin-coated wells and incubated for 18 hours at 37°C. The plate was then washed six times with PBS containing 0.05% Tween 20. Subsequently, 100 µL of horseradish peroxidase-conjugated peanut agglutinin lectin (PNA-HRPO, Sigma-Aldrich) in MES buffer (pH 6.5) with CaCl2 and 1% bovine serum albumin was added to each well and incubated for 2 hours at room temperature in the dark. Following this, the plate was washed six times and developed with 1-Step TMB ELISA substrate solutions (Thermo Fisher Scientific). The absorbance was measured at 450 nm using a SpectraMax M2 microplate reader (Molecular Devices). Data points were analyzed using Prism software, and the 50% inhibition concentration (IC_50_) was determined as the concentration at which 50% of the neuraminidase activity was inhibited, compared to the negative control.

## Data Availability

The cryoEM maps for influenza HA and NA in Fig. 9 and 10 were downloaded from https://www.rcsb.org/ with accession code 6Q23 for NA and 3LZG for HA.

## References

[1] Wu NC, Wilson IA. 2020. Influenza hemagglutinin structures and antibody recognition. Cold Spring Harb Perspect Med 10:a038778. doi:10.1101/cshperspect.a038778 31871236 PMC7397844

[2] Krammer F. 2019. The human antibody response to influenza A virus infection and vaccination. Nat Rev Immunol 19:383–397. doi:10.1038/s41577-019-0143-6 30837674

[3] Lei R, Kim W, Lv H, Mou Z, Scherm MJ, Schmitz AJ, Turner JS, Tan TJC, Wang Y, Ouyang WO, Liang W, Rivera-Cardona J, Teo C, Graham CS, Brooke CB, Presti RM, Mok CKP, Krammer F, Dai X, Ellebedy AH, Wu NC. 2023. Leveraging vaccination-induced protective antibodies to define conserved epitopes on influenza N2 neuraminidase. Immunity 56:2621–2634. doi:10.1016/j.immuni.2023.10.005 37967533 PMC10655865

[4] Stadlbauer D, Zhu X, McMahon M, Turner JS, Wohlbold TJ, Schmitz AJ, Strohmeier S, Yu W, Nachbagauer R, Mudd PA, Wilson IA, Ellebedy AH, Krammer F. 2019. Broadly protective human antibodies that target the active site of influenza virus neuraminidase. Science 366:499–504. doi:10.1126/science.aay0678 31649200 PMC7105897

[5] Momont C, Dang HV, Zatta F, Hauser K, Wang C, di Iulio J, Minola A, Czudnochowski N, De Marco A, Branch K, et al.. 2023. A pan-influenza antibody inhibiting neuraminidase via receptor mimicry. Nature New Biol 618:590–597. doi:10.1038/s41586-023-06136-y PMC10266979 37258672

[6] Henry C, Palm A-KE, Krammer F, Wilson PC. 2018. From original antigenic sin to the universal influenza virus vaccine. Trends Immunol 39:70–79. doi:10.1016/j.it.2017.08.003 28867526 PMC5748348

[7] Francis Jr T. 1960. On the doctrine of original antigenic sin. Proc Am Philos Soc 104:572–578. https://www.jstor.org/stable/985534?seq=7.

[8] Lv H, So RTY, Teo QW, Yuan M, Liu H, Lee C-CD, Yip GK, Ng WW, Wilson IA, Peiris M, Wu NC, Mok CKP. 2022. Neutralizing antibody response to Sarbecovirus is delayed in sequential heterologous immunization. Viruses 14:1382. doi:10.3390/v14071382 35891363 PMC9318566

[9] Koutsakos M, Ellebedy AH. 2023. Immunological imprinting: understanding COVID-19. Immunity 56:909–913. doi:10.1016/j.immuni.2023.04.012 37105169 PMC10113596

[10] Halstead SB, Rojanasuphot S, Sangkawibha N. 1983. Original antigenic sin in dengue. Am J Trop Med Hyg 32:154–156. doi:10.4269/ajtmh.1983.32.154 6824120

[11] Rothman AL. 2011. Immunity to dengue virus: a tale of original antigenic sin and tropical cytokine storms. Nat Rev Immunol 11:532–543. doi:10.1038/nri3014 21760609

[12] Johnston TS, Li SH, Painter MM, Atkinson RK, Douek NR, Reeg DB, Douek DC, Wherry EJ, Hensley SE. 2024. Immunological imprinting shapes the specificity of human antibody responses against SARS-CoV-2 variants. Immunity 57:912–925. doi:10.1016/j.immuni.2024.02.017 38490198 PMC13084757

[13] Tortorici MA, Addetia A, Seo AJ, Brown J, Sprouse K, Logue J, Clark E, Franko N, Chu H, Veesler D. 2024. Persistent immune imprinting occurs after vaccination with the COVID-19 XBB.1.5 mRNA booster in humans. Immunity 57:904–911. doi:10.1016/j.immuni.2024.02.016 38490197 PMC12360627

[14] Kucharski AJ, Lessler J, Read JM, Zhu H, Jiang CQ, Guan Y, Cummings DAT, Riley S. 2015. Estimating the life course of influenza A(H3N2) antibody responses from cross-sectional data. PLoS Biol 13:e1002082. doi:10.1371/journal.pbio.1002082 25734701 PMC4348415

[15] Fonville JM, Wilks SH, James SL, Fox A, Ventresca M, Aban M, Xue L, Jones TC, Le NMH, Pham QT, et al.. 2014. Antibody landscapes after influenza virus infection or vaccination. Science 346:996–1000. doi:10.1126/science.1256427 25414313 PMC4246172

[16] Cobey S, Hensley SE. 2017. Immune history and influenza virus susceptibility. Curr Opin Virol 22:105–111. doi:10.1016/j.coviro.2016.12.004 28088686 PMC5467731

[17] Dawood FS, Iuliano AD, Reed C, Meltzer MI, Shay DK, Cheng P-Y, Bandaranayake D, Breiman RF, Brooks WA, Buchy P, et al.. 2012. Estimated global mortality associated with the first 12 months of 2009 pandemic influenza A H1N1 virus circulation: a modelling study. Lancet Infect Dis 12:687–695. doi:10.1016/S1473-3099(12)70121-4 22738893

[18] Nguyen AM, Noymer A. 2013. Influenza mortality in the United States, 2009 pandemic: burden, timing and age distribution. PLoS One 8:e64198. doi:10.1371/journal.pone.0064198 23717567 PMC3661470

[19] Gouma S, Kim K, Weirick ME, Gumina ME, Branche A, Topham DJ, Martin ET, Monto AS, Cobey S, Hensley SE. 2020. Middle-aged individuals may be in a perpetual state of H3N2 influenza virus susceptibility. Nat Commun 11:4566. doi:10.1038/s41467-020-18465-x 32917903 PMC7486384

[20] Arevalo CP, Le Sage V, Bolton MJ, Eilola T, Jones JE, Kormuth KA, Nturibi E, Balmaseda A, Gordon A, Lakdawala SS, Hensley SE. 2020. Original antigenic sin priming of influenza virus hemagglutinin stalk antibodies. Proc Natl Acad Sci U S A 117:17221–17227. doi:10.1073/pnas.1920321117 32631992 PMC7382271

[21] McCarthy KR, Von Holle TA, Sutherland LL, Oguin TH III, Sempowski GD, Harrison SC, Moody MA. 2021. Differential immune imprinting by influenza virus vaccination and infection in nonhuman primates. Proc Natl Acad Sci USA 118. doi:10.1073/pnas.2026752118 PMC8201799 34074774

[22] Gostic KM, Bridge R, Brady S, Viboud C, Worobey M, Lloyd-Smith JO. 2019. Childhood immune imprinting to influenza A shapes birth year-specific risk during seasonal H1N1 and H3N2 epidemics. PLoS Pathog 15:e1008109. doi:10.1371/journal.ppat.1008109 31856206 PMC6922319

[23] Yewdell JW, Santos JJS. 2021. Original antigenic sin: how original? How sinful? Cold Spring Harb Perspect Med 11:a038786. doi:10.1101/cshperspect.a038786 31964645 PMC8091961

[24] Gostic KM, Ambrose M, Worobey M, Lloyd-Smith JO. 2016. Potent protection against H5N1 and H7N9 influenza via childhood hemagglutinin imprinting. Science 354:722–726. doi:10.1126/science.aag1322 27846599 PMC5134739

[25] Rajendran M, Nachbagauer R, Ermler ME, Bunduc P, Amanat F, Izikson R, Cox M, Palese P, Eichelberger M, Krammer F. 2017. Analysis of anti-influenza virus neuraminidase antibodies in children, adults, and the elderly by ELISA and enzyme inhibition: evidence for original antigenic sin. MBio 8:e02281-16. doi:10.1128/mBio.02281-16 28325769 PMC5362038

[26] Daulagala P, Mann BR, Leung K, Lau EHY, Yung L, Lei R, Nizami SIN, Wu JT, Chiu SS, Daniels RS, Wu NC, Wentworth D, Peiris M, Yen HL. 2023. Imprinted anti-hemagglutinin and anti-neuraminidase antibody responses after childhood infections of A(H1N1) and A(H1N1)pdm09 influenza viruses. MBio 14:e0008423. doi:10.1128/mbio.00084-23 37070986 PMC10294682

[27] Anderson CS, McCall PR, Stern HA, Yang H, Topham DJ. 2018. Antigenic cartography of H1N1 influenza viruses using sequence-based antigenic distance calculation. BMC Bioinformatics 19:51. doi:10.1186/s12859-018-2042-4 29433425 PMC5809904

[28] Neumann G, Fujii K, Kino Y, Kawaoka Y. 2005. An improved reverse genetics system for influenza A virus generation and its implications for vaccine production. Proc Natl Acad Sci U S A 102:16825–16829. doi:10.1073/pnas.0505587102 16267134 PMC1283806

[29] Sandbulte MR, Westgeest KB, Gao J, Xu X, Klimov AI, Russell CA, Burke DF, Smith DJ, Fouchier RAM, Eichelberger MC. 2011. Discordant antigenic drift of neuraminidase and hemagglutinin in H1N1 and H3N2 influenza viruses. Proc Natl Acad Sci U S A 108:20748–20753. doi:10.1073/pnas.1113801108 22143798 PMC3251064

[30] Kilbourne ED, Johansson BE, Grajower B. 1990. Independent and disparate evolution in nature of influenza A virus hemagglutinin and neuraminidase glycoproteins. Proc Natl Acad Sci U S A 87:786–790. doi:10.1073/pnas.87.2.786 2300562 PMC53351

[31] Impagliazzo A, Milder F, Kuipers H, Wagner MV, Zhu X, Hoffman RMB, van Meersbergen R, Huizingh J, Wanningen P, Verspuij J, et al.. 2015. A stable trimeric influenza hemagglutinin stem as A broadly protective immunogen. Science 349:1301–1306. doi:10.1126/science.aac7263 26303961

[32] Corti D, Voss J, Gamblin SJ, Codoni G, Macagno A, Jarrossay D, Vachieri SG, Pinna D, Minola A, Vanzetta F, Silacci C, Fernandez-Rodriguez BM, Agatic G, Bianchi S, Giacchetto-Sasselli I, Calder L, Sallusto F, Collins P, Haire LF, Temperton N, Langedijk JPM, Skehel JJ, Lanzavecchia A. 2011. A neutralizing antibody selected from plasma cells that binds to group 1 and group 2 influenza A hemagglutinins. Science 333:850–856. doi:10.1126/science.1205669 21798894

[33] Colman PM, Varghese JN, Laver WG. 1983. Structure of the catalytic and antigenic sites in influenza virus neuraminidase. Nature New Biol 303:41–44. doi:10.1038/303041a0 6188957

[34] Xiong FF, Liu XY, Gao FX, Luo J, Duan P, Tan WS, Chen Z. 2020. Protective efficacy of anti-neuraminidase monoclonal antibodies against H7N9 influenza virus infection. Emerg Microbes Infect 9:78–87. doi:10.1080/22221751.2019.1708214 31894728 PMC6968527

[35] O’Donnell CD, Wright A, Vogel L, Boonnak K, Treanor JJ, Subbarao K. 2014. Humans and ferrets with prior H1N1 influenza virus infections do not exhibit evidence of original antigenic sin after infection or vaccination with the 2009 pandemic H1N1 influenza virus. Clin Vaccine Immunol 21:737–746. doi:10.1128/CVI.00790-13 24648486 PMC4018878

[36] Liu STH, Behzadi MA, Sun W, Freyn AW, Liu WC, Broecker F, Albrecht RA, Bouvier NM, Simon V, Nachbagauer R, Krammer F, Palese P. 2018. Antigenic sites in influenza H1 hemagglutinin display species-specific immunodominance. J Clin Invest 128:4992–4996. doi:10.1172/JCI122895 30188868 PMC6205383

