# Correction for Lv et al., “Differential antigenic imprinting effects between influenza H1N1 hemagglutinin and neuraminidase in a mouse model”

**DOI:** 10.1128/jvi.00783-25

**Published:** 2025-06-12

**Authors:** Huibin Lv, Qi Wen Teo, Chang-Chun D. Lee, Weiwen Liang, Danbi Choi, Kevin J. Mao, Madison R. Ardagh, Akshita B. Gopal, Arjun Mehta, Matt Szlembarski, Roberto Bruzzone, Ian A. Wilson, Nicholas C. Wu, Chris K. P. Mok

## AUTHOR CORRECTION

Volume 99, no. 1, e01695-24, 2025, https://doi.org/10.1128/jvi.01695-24. The following statement should be removed from Acknowledgments: “and NIAID Centers of Excellence for Influenza Research and Response 75N93021C00015 (I.A.W., N.C.W.).”

